# MRI detected mass decrease in patients with amyloidosis after treatment with green tea: a one year follow-up study

**DOI:** 10.1186/1532-429X-14-S1-P173

**Published:** 2012-02-01

**Authors:** Sebastian Buss, Arnt Kristen, Stephanie Lehrke, Hugo A Katus

**Affiliations:** 1 Department of Cardiology, University of Heidelberg, Heidelberg, Germany

## Background

Amyloidosis (AM) is a disease complex that results from extra-cellular protein deposition of uniquely twisted and insoluble beta pleated sheet fibrils that can be found in several acquired or hereditary AM forms. Clinically apparent cardiac involvement can be seen in about 30% of patients resulting in a restrictive cardiomyopathy, which is the most common cause of death in these patients. Recently, there was compelling evidence that polyphenol epigallo-catechin-3-gallate (EGCG), a chemical component of regular green tea, induces aggregation products that do not polymerize into fibrils. Cardiac MRI is the reference standard for measurement of cardiac function, myocardial mass and cardiac remodeling but has not been used for longitudinal follow-up of AM patients treated with EGCG.

We hypothesized that cardiac MRI is able to detect changes in cardiac function, myocardial mass and LV remodeling in AM patients being treated with EGCG for 12 months.

## Methods

In a twelve-month follow-up, we studied 12 patients (70±7 ys, 2 women) with acquired or genetic forms of AM using a whole body 1.5T CMR scanner (Philips Achieva). Vector-ECG gated short axis, two and four chamber cine slices with parallel image acquisition covering the entire left ventricle (LV) were acquired using a regular SSFP sequence. LV volumes and myocardial mass were acquired by manual drawing of endo- and epicardial borders. In a blinded fashion, two observers at different time-points assessed twice left ventricular (LV) end-diastolic and systolic volumes (LVEDV, LVESV), stroke volume (LVSV), ejection fraction (LVEF) and longitudinal shortening. P<0.05 was considered statistically significant.

## Results

Results are shown in Table 1. After twelve months, there was a highly significant reduction in LV mass, a significant increase in EDV, whereas EF, ESV and SV, although marginal, did not reach statistical significance.

**Table 1:** 

MR Parameters	Pre EGCG	Std.	Post EGCG	Std.	Significance
LV mass (g)	181	48	158	42	<0.01
LV EF (%)	57	14	57	11	n.s.
LVEDV (ml)	170	57	175	50	0.05
LVESV (ml)	77	44	79	48	0.07
LVSV (ml)	94	29	96	11	0.08

## Conclusions

In a small study cohort of AM patients treated with EGCG, our results provide preliminary evidence that after a twelve month period of treatment, myocardial mass was significantly reduced, although myocardial function was primarily unaffected. The molecular mechanisms- whether EGCG can elutriate existing extracellular fibrils or whether it modifies the deficient proteins avoiding the extracellular precipitation- as well as the clinical relevance regarding modified risk stratification, future cardiac events and event-free survival warrant further investigation in a much larger patient cohort.

## Funding

None.

